# Deep learning enables automated scoring of liver fibrosis stages

**DOI:** 10.1038/s41598-018-34300-2

**Published:** 2018-10-30

**Authors:** Yang Yu, Jiahao Wang, Chan Way Ng, Yukun Ma, Shupei Mo, Eliza Li Shan Fong, Jiangwa Xing, Ziwei Song, Yufei Xie, Ke Si, Aileen Wee, Roy E. Welsch, Peter T. C. So, Hanry Yu

**Affiliations:** 1 Institute of Bioengineering and Nanotechnology, Agency for Science, Technology and Research (A*STAR), Singapore, 138669 Singapore; 2 Department of Physiology, Yong Loo Lin School of Medicine, National University of Singapore, Singapore, 117597 Singapore; 3 BioSystems and Micromechanics (BioSyM), Singapore-MIT Alliance for Research and Technology, Singapore, 138602 Singapore; 4 Institute of Neuroscience, Department of Neurobiology, Key Laboratory of Medical Neurobiology of the Ministry of Health of China, Zhejiang Province Key Laboratory of Neurobiology, School of Medicine, Zhejiang University, Zhejiang, 310058 China; 5 NUS Graduate School of Integrative Sciences and Engineering, National University of Singapore, Singapore, 117411 Singapore; 6 Mechanobiology Institute, National University of Singapore, Singapore, 117411 Singapore; 7 Department of Biomedical Engineering, National University of Singapore, Singapore, 117583 Singapore; 8 Duke-NUS Graduate Medical School Singapore, National University of Singapore, Singapore, 169857 Singapore; 9 State Key Laboratory of Modern Optical Instrumentation, College of Optical Science and Engineering, Zhejiang University, Zhejiang, 310027 China; 10 Department of Pathology, National University Hospital, Singapore, 119074 Singapore; 11 Department of Pathology, Yong Loo Lin School of Medicine, National University of Singapore, Singapore, 119074 Singapore; 12 Sloan School of Management, Massachusetts Institute of Technology, Cambridge, MA 02139 USA; 13 Center for Statistics and Data Science, Massachusetts Institute of Technology, Cambridge, MA 02139 USA; 14 Department of Mechanical Engineering, Massachusetts Institute of Technology, Cambridge, MA 02139 USA; 15 Department of Biological Engineering, Massachusetts Institute of Technology, Cambridge, MA 02139 USA; 16 Confocal Microscopy Unit & Flow Cytometry Laboratory, National University Health System, Singapore, 119228 Singapore; 17 Gastroenterology Department, Nanfang Hospital, Southern Medical University, Guangzhou, 510515 China

## Abstract

Current liver fibrosis scoring by computer-assisted image analytics is not fully automated as it requires manual preprocessing (segmentation and feature extraction) typically based on domain knowledge in liver pathology. Deep learning-based algorithms can potentially classify these images without the need for preprocessing through learning from a large dataset of images. We investigated the performance of classification models built using a deep learning-based algorithm pre-trained using multiple sources of images to score liver fibrosis and compared them against conventional non-deep learning-based algorithms - artificial neural networks (ANN), multinomial logistic regression (MLR), support vector machines (SVM) and random forests (RF). Automated feature classification and fibrosis scoring were achieved by using a transfer learning-based deep learning network, AlexNet-Convolutional Neural Networks (CNN), with balanced area under receiver operating characteristic (AUROC) values of up to 0.85–0.95 versus ANN (AUROC of up to 0.87–1.00), MLR (AUROC of up to 0.73–1.00), SVM (AUROC of up to 0.69–0.99) and RF (AUROC of up to 0.94–0.99). Results indicate that a deep learning-based algorithm with transfer learning enables the construction of a fully automated and accurate prediction model for scoring liver fibrosis stages that is comparable to other conventional non-deep learning-based algorithms that are not fully automated.

## Introduction

Machine-learning is popular for the prediction, classification and assessment of liver fibrosis/cirrhosis, and response to therapy in hepatitis B and C patients^1–3^. At present, histopathological examination of liver biopsy samples remains as the ‘gold standard’ for liver fibrosis assessment. Histopathological features such as excessive accumulation of extracellular matrix (particularly collagen) and/or parenchymal extinction^4^ are incorporated into descriptive or semi-quantitative scoring systems for disease staging. Examples include the Knodell histological activity index (HAI)^5^, Scheuer^6^, Ishak^1^, Metavir^7^, and Ishak-modified systems^8^. However, these scoring systems are inherently semi-quantitative and subject to intra- and inter-observer variability.

To overcome these limitations, we and others have previously developed systems using machine learning-based algorithms to build diagnostic tools for improved liver fibrosis quantification and scoring^9–11^. These algorithms include artificial neural networks (ANN), multinomial logistic regression (MLR), support vector machines (SVM), and random forests (RF). However, the relative accuracy of these algorithms for liver fibrosis assessment and scoring remains unknown. Furthermore, besides having a limited number of extracted features, these algorithms require manual segmentation and feature extraction from the images prior to modeling and classification^12^. Therefore, we sought to develop fully automated algorithms for staging liver fibrosis.

Deep learning-based algorithms provide a powerful framework for automatic feature generation and image classification using magnetic resonance imaging (MRI) and computerized tomography (CT) images on liver fibrosis^13,14^. Such algorithms eliminate the need for manual segmentation and feature extraction from the images. However, they demand the use of a large training dataset and biopsy sample images typically do not meet this need. Based on transfer learning being a variant of the typical deep learning-based algorithms - in that the neural network is pre-trained by a very large number of training datasets worldwide using weakly or even irrelevant image sources^15,16^ - we hypothesized that the pre-trained deep learning neural network in transfer learning approach can accurately stage liver fibrosis in a fully automated manner. Here, we validated our hypothesis by inheriting and adapting the most studied seven-layered AlexNet^17^ algorithm for computer-aided liver fibrosis scoring. We compared the accuracy of this deep learning-based algorithm with other semi-automated machine learning algorithms – ANN, MLR, SVM and RF. Validation results showed that the deep learning-based algorithm by transfer learning approach can fully automate the scoring of liver fibrosis stages, with accuracy similar to conventional ANN, non-linear MLR, linear SVM and feature-ranking based RF algorithms. It is potentially a powerful tool that can aid disease diagnosis even with limited numbers of biopsy samples or clinical information.

## Materials and Methods

### Tissue preparation

Male Wistar rats (average weight of 220 g) were housed 2 per cage in the Biological Resource Centre (BRC) of Biopolis, Agency for Science, Technology and Research (A*STAR) with free access to laboratory chow and water in a 12:12 h light/dark schedule. The Institutional Animal Care and Use Committee (IACUC, Biological Resource Centre, A*STAR) approved all animal-related experiments and all methods were performed in accordance with the relevant guidelines and regulations. A total of 25 rats were randomly separated into a group of 21 for thioacetamide (TAA) treatment, and a group of 4 as controls. The 21 rats in the TAA-treated group were sacrificed at time-points of 4, 6, 7, 8, 10 and 12 weeks. Another 4 rats in the control group were sacrificed at week 0 without treatment. Cardiac perfusion with 4% paraformaldehyde was performed to flush out blood cells and the liver was fixed before harvesting.

### Collagen staining and pathological scoring

4 biopsy samples were taken from each rat liver. They were formalin-fixed, paraffin-embedded, cut into 5 µm sections and stained for histological assessment. A total of 100 slides from 25 paraffin blocks were stained with Sirius Red (SR) stain kit (Picro Sirius Red Stain Kit, ab150681, Abcam). Each SR-stained sample was blinded and scored by a pathologist to reduce any bias using the Metavir scoring system^7^. In the Metavir system, liver fibrosis was classified into five stages from F0 to F4 according to the severity of fibrosis: no fibrosis, fibrous portal expansion, few bridges or septa, numerous bridges or septa, and cirrhosis^18^. The number of samples that were categorized into each stage was: 20 F0, 20 F1, 20 F2, 20 F3 and 20 F4.

### Image acquisition

Another 4 paired biopsy samples were taken from the previously processed 25 paraffin blocks for imaging using Second Harmonic Generation (SHG) microscopy. A total of 100 paired slides from 25 paraffin blocks were used and SHG images were acquired using the Olympus IX81 system. The laser was tuned to 810 nm to excite the samples and SHG signals were recorded at 405 nm using a 20X objective lens. A nine-by-seven multi-tile image was acquired for every slide with a final image size of 12 mm^2^ (4 × 3 mm).

### Image pre-processing for collagen content

All raw images acquired from SHG microscopy were imported into the MATLAB Image Processing Toolbox (Mathworks) for further processing. The resulting gray-scale images were first adjusted by contrast enhancement^19^ and then transformed into binary images using adaptive-thresholding^9–11^. To fully reproduce the distribution of collagen fibers and eliminate any background signal, morphological closing was then performed to smooth the binary mask of the collagen and segments with less than 5 pixels were removed^9–11^.

### Feature extraction and quantification for collagen content

Total collagen content and previously described collagen features, including collagen fiber morphology^20,21^ and collagen fiber connectivity-related texture measurements^22^, were investigated. A total of 21 morphological features specific to tubular-shaped objects including length, width, orientation, cross-link spaces and cross-link density, etc. and 109 textural features of both gray-level co-occurrence matrix (GLCM) and transform-based pattern including contrast, correlation, average, variation, entropy etc. from the occurrence matrix and Fourier, Gabor, and wavelet transformation were extracted from the processed SHG images.

### Model construction for deep learning-based algorithms

For deep learning, a pre-trained AlexNet-CNN network was used for training and testing purposes. This network was made up of 1 input layer, 7 hidden layers (5 convolution layers and 2 fully connected layers) and 1 output layer using batch stochastic gradient descent, with specific values for momentum and weight decay^17^. The input and output layers of the original AlexNet-CNN network were replaced accordingly for liver fibrosis assessment as previously reported^15,16^, where the processed SHG images were first resized and duplicated to 224 × 224 × 3 pixels to fit into the model as input images. There were 2 max pooling layers of size 2 × 2 pixels after the first and second convolution layer where the size and number of filters are 11 × 11 × 3 pixels, 96 and 5 × 5 × 96 pixels, 256, respectively. Another 3 convolution layers where the size and number of filters are 3 × 3 × 256 pixels, 384 and 3 × 3 × 384 pixels, 384 and 3 × 3 × 384 pixels, 256, respectively were also implemented before the third max pooling layer. Layers 6 and 7 are 4096-dimension fully connected layers where the input matrix is transformed into a vector for Softmax activation function through General MATRIX Vector Multiply (GEMV) approach. The final output layer contained 5 possible outcomes corresponding to the 5 liver fibrosis stages from F0 to F4 using the Metavir scoring.

### Model construction for non-deep learning-based algorithms

ANN, MLR, SVM and RF-based algorithms were applied for the training and testing datasets with pre-processed images and selected features to test the sample classification. The leave-one-out cross validation rule was applied.

For ANN, various combinations of layers and nodes a) 1 hidden layer with 10 nodes; b) 1 hidden layer with 20 nodes and c) 2 hidden layers with 20 nodes each and feed-forward neural network were established for the analysis using a back-propagation orientated gradient descent training approach to optimize the loss.

For MLR, the coefficients of the predicted probability were calculated using the logistic function, g, the logit (log odds):

$${\rm{g}}({\rm{p}}({\rm{x}}))=\,\mathrm{ln}(\frac{p(x)}{1-p(x)})={\beta }_{0}+{\beta }_{1}{x}_{1}+\ldots +{\beta }_{n}{x}_{n}$$

The coefficients ${\beta }_{0}$, ${\beta }_{1}$, …${\beta }_{n}$ were estimated with the maximum likelihood method.

For SVM, a hyperplane was constructed for optimally and correctly classifying the images into fibrosis stages based on the feature values in the m-dimensional space. The radial basis function (RBF) kernel was used as the distance measurement between adjacent subject vectors ${x}_{1}$ and ${x}_{2}$, i.e.,

$${\rm{K}}({x}_{1},{x}_{2})=\exp (-\frac{({x}_{1}-{{x}_{2})}^{2}}{2{\sigma }^{2}})$$

$\sigma$ controls the width of the kernel.

For RF, the training algorithm applies the general techniques of bootstrap aggregating, or bagging, to trees learners where 100 decision tree bagging with random sample replacement was used to generate the classification results and the majority vote in the case of classification trees was used for final predictions. The core algorithm for building decision trees called ID3 by J. R. Quinlan employs a top-down, greedy search through the space of possible branches with no backtracking. ID3 uses Entropy and Information Gain to construct a decision tree^23^.

### Statistical analysis

Kruskal-Wallis (KW) tests were used to assess the performance of various classification models for their prediction capabilities in SPSS (IBM Corporation). Post-hoc analysis was performed to verify significant KW results and compare the deep learning method against other non-deep learning methods using Wilcoxon–Mann–Whitney tests and Bonferroni correction methods. Since there were 5 classification models for each comparison, 10 comparisons using Wilcoxon–Mann–Whitney test were generated. According to Bonferroni correction, the adjusted critical p-value for 0.05 significance would be 0.05 divided by the total number of comparisons, or 0.005 (0.05/10). Thus, the acquired p-values by Wilcoxon–Mann–Whitney tests were compared with the adjusted critical p-value 0.005 and the p < 0.005 criterion was used.

## Results

### Correlation of SHG microscopy-based quantification of collagen distribution with pathological scoring

**Figure 1:**
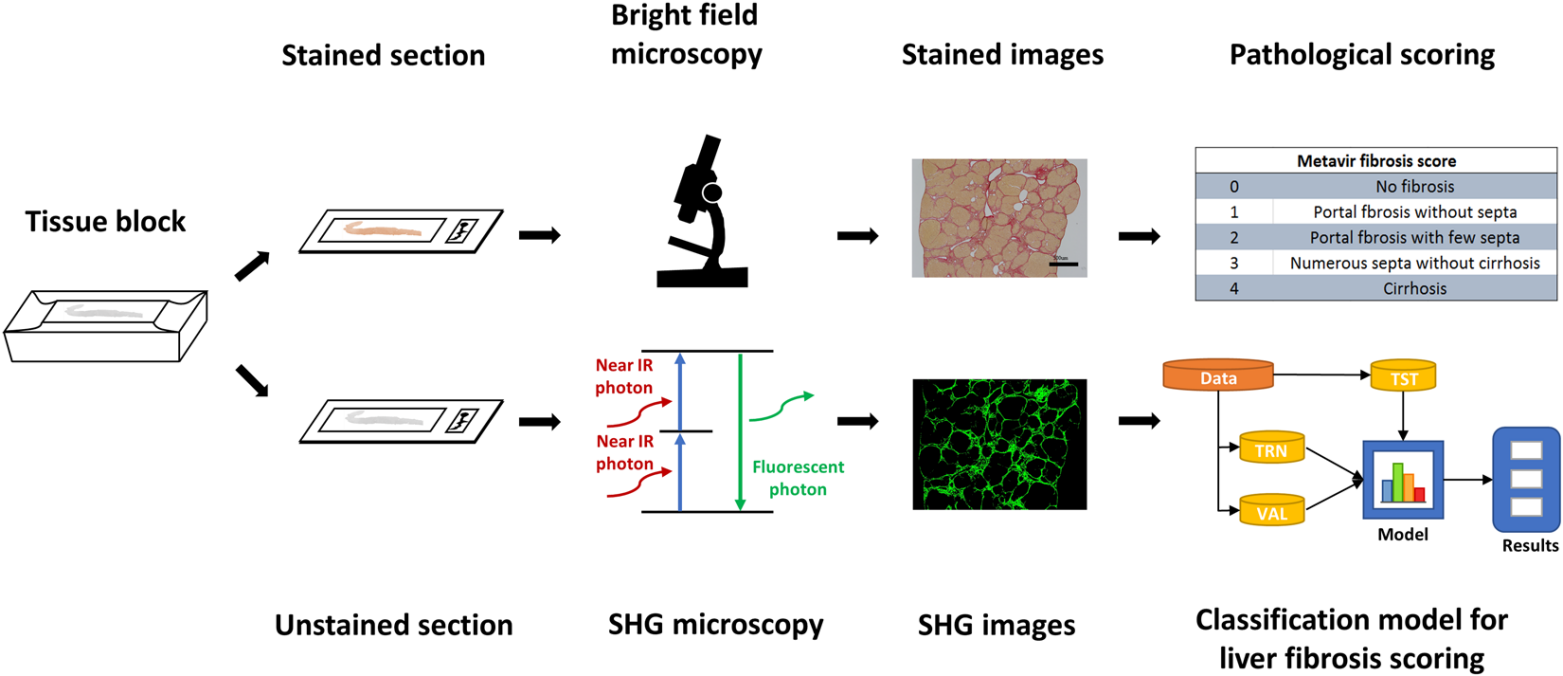
Schematic of study outline. Tissues were fixed, dehydrated, embedded in paraffin and sectioned into 2 consecutive slides. One was stained with Sirius Red (SR) for histological assessment by a pathologist using a conventional bright field microscope. The other was imaged using second harmonic generation (SHG) microscopy to generate images for subsequent use in developing a computer-aided classification model. TRN: training group, VAL: validation group, TST: testing group.

**Figure 2:**
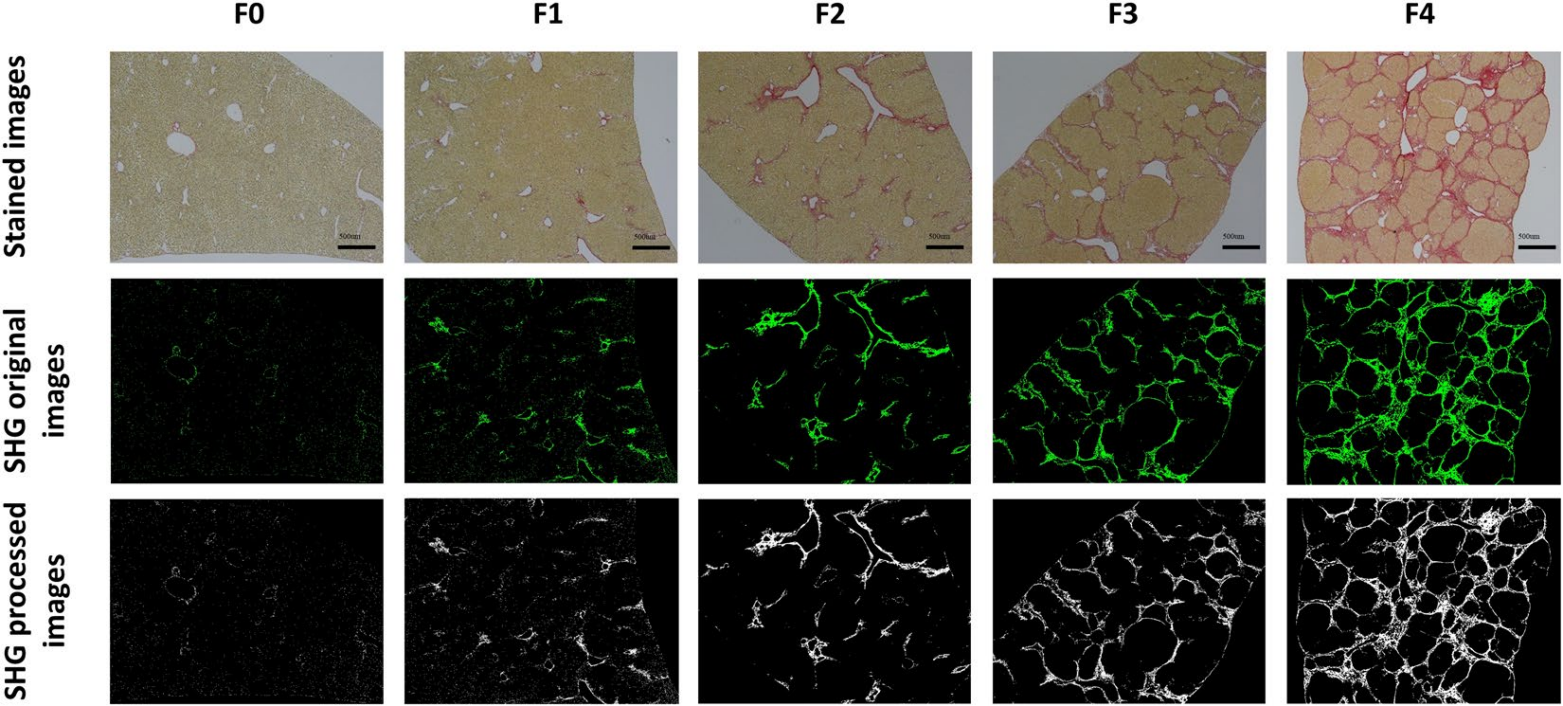
Representative images of SR-stained samples, SHG original images and SHG processed images at various stages of liver fibrosis.

To quantify collagen distribution in liver fibrosis of different stages, we acquired 100 liver section images of TAA-induced fibrotic rat livers using SHG microscopy. Liver samples were also stained with Sirius Red (SR) for histopathological review and scored by a pathologist (Table S1) using the METAVIR scoring system (Fig. 1). Qualitatively, SHG microscopy yielded images with higher collagen contrast as compared to the SR-stained images. To monitor collagen distribution during disease progression, we performed image processing for collagen tracing. The image-processing algorithm reinforces the collagen signal by using adaptive thresholding on the gray scale image, identifying morphological/textural changes in the binary image and removing noise by multiplying the tissue binary image. Figure 2 shows representative original and processed SHG images compared to SR-stained images at various stages of liver fibrosis.

### Development for various supervised classification models

**Figure 3:**
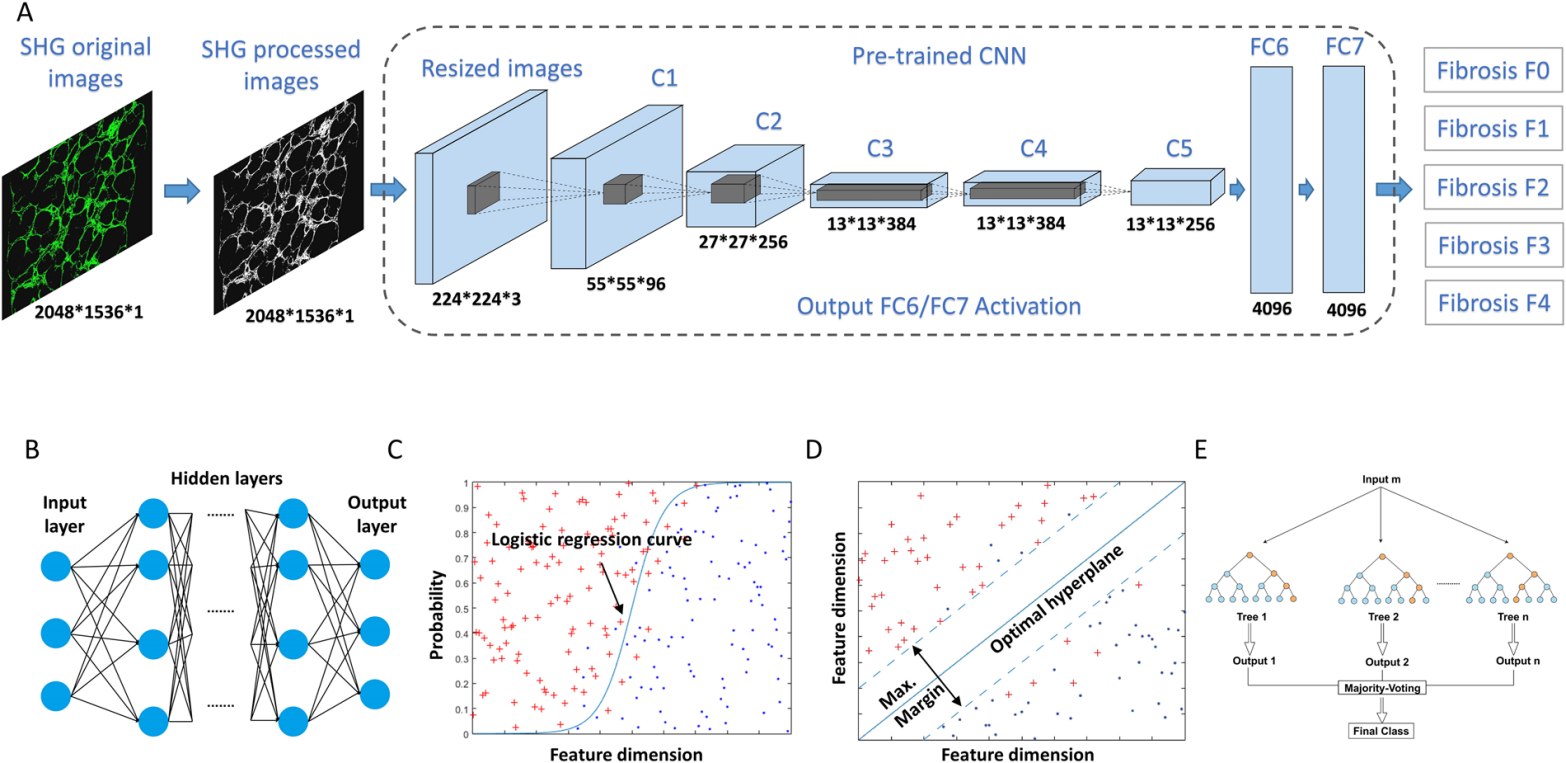
The architecture and of (**A**) deep learning via convolutional neuron networks (CNN) using pretrained 7-layered AlexNet and non-deep learning via (**B**) Artificial Neural Networks (ANN), (**C**) Multinomial Logistic Regression (MLR), (**D**) Support Vector Machines (SVM) and Random Forests (RF) for computer aided liver fibrosis scoring. SHG processed images were resized and duplicated to be used as input for deep learning-based algorithm, C1-C5: convolution layer, FC6-FC7: fully connected layer. Extracted morphological or/and textural features were used as input for non-deep learning-based algorithms.

**Figure 4:**
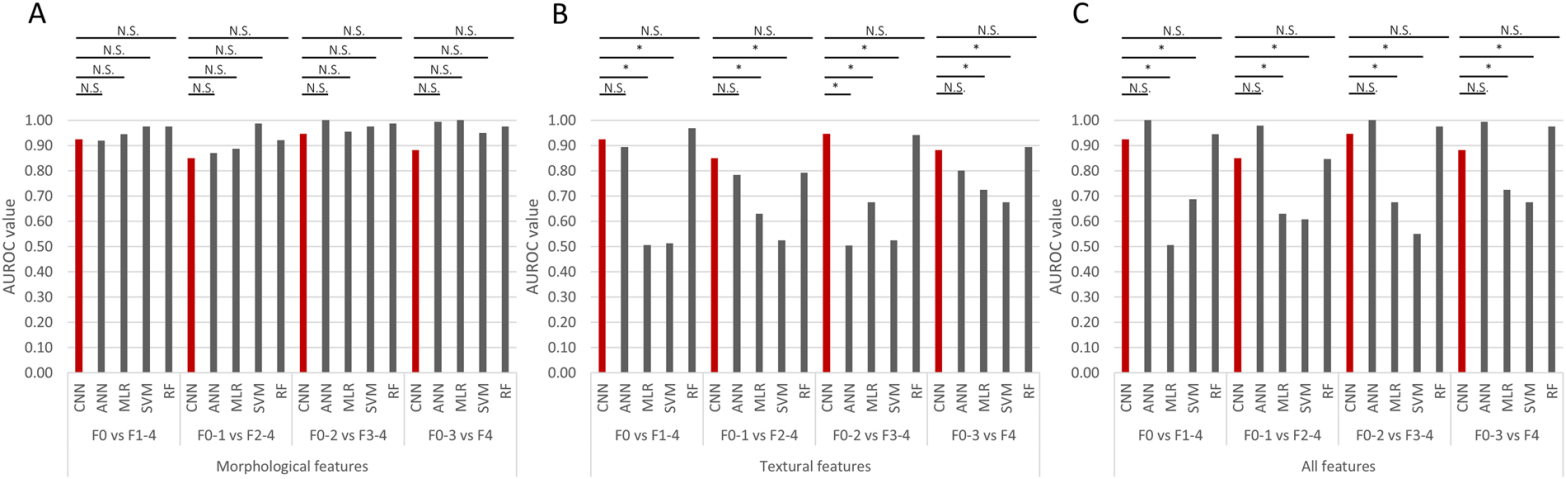
The area under receiver operating characteristic (AUROC) values for various classification models from AlexNet-Convolutional Neuron Networks (CNN), conventional Artificial Neural Networks (ANN), non-linear Multinomial Logistic Regression (MLR), linear Support Vector Machines (SVM) and feature-ranking based Random Forests (RF) algorithms. AUROC was evaluated for CNN and ANN, MLR, SVM and RF with (**A**) morphological features (Features 1–21), (**B**) textural features (Features 21–130) and (**C**) both morphological and textural features (Feature 1–130). N.S.: non-significant difference, *adjusted p value is less than 0.005.

Based on the pathological scoring of the METAVIR fibrosis stages, we used different supervised machine learning-based algorithms to generate classification models, namely deep learning CNN, ANN, MLR, SVM, and RF models (Fig. 3). All samples were investigated using a leave-one-out (LOO) rule for validation purposes due to the limited number of samples in the dataset. For deep learning-based algorithms, automatic feature extraction was performed during the convolution and max pooling process. For ANN, MLR, SVM and RF-based algorithms, we quantitatively measured the extent of liver fibrosis by examining the morphology and textural features of collagen fibers. 21 morphological features (Features 1–21) and 109 textural features [based on entropy (Feature 22), gray-level co-occurrence matrix (GLCM) (Features 23–34), Fourier’s transform (Features 35–40), wavelet decomposition (Features 41–100) and magnitude of convolution (Features 101–130)] were analyzed to characterize collagen quantity and distribution pattern. Table 1 lists the features that were extracted from the SHG-generated images. Morphological features (Features 1–21) were first used for model fitting. Subsequently, textural features (Features 22–130) were used for further analysis. Finally, morphological and textural features (Features 1–130) were combined. As expected, with more and some less important features being added into the model, some of the algorithms failed to correctly classify the various stages of fibrosis progression such as MLR and SVM. This is because an increase in the number of extracted features being incorporated into the model typically decrease the diagnostic power of those linear and simple classifiers without weighting on different features or feature selection step. The receiver operating characteristic (ROC) curve was used to assess the performance of the classification model with samples from the test cohort (Fig. 4). Details on the ROC curves and AUROC values can be found in Figs S1 and S2. Table 2 shows sensitivity, specificity, positive predictive values (PPV) and negative predictive values (NPV) for all classification models.

**Table 1:** Extracted morphological and textural features of collagen fibers from SHG processed images.

No.	Feature Descriptions
**Morphological Features**	
1	Total number of collagen fibers
2–3	Median and variance of fiber orientation
4–6	Median, total and variance of fiber length
7–9	Median, total and variance of fiber width
10	Total perimeter of collagen fibers
11–14	No. of long & short and thick & thin fibers
15–16	Ratio of short/long fibers and thin/thick fibers
17–19	Median, total and variance of fiber area
20	Collagen mean intensity
21	Fiber proportionate area (CPA)
**Texture Features**	
22	Entropy
23–34	Contrast, correlation, energy and homogeneity from the GLCM given three different pixel distances at two, four, eight pixels
35–40	Energy, entropy, mean, standard deviation, third moment and fourth moment of the coefficients from Fouriers transform
41–100	Energy, entropy, mean, standard deviation, third moment and fourth moment of the wavelet decomposition coefficients from ten sub-images generated by Daubechies wavelet transform
101–130	Energy, entropy, mean, standard deviation, third moment and fourth moment of the magnitude of the convolution over the image with Gabor filters at five scales

**Table 2:** The performance evaluation for CNN, ANN, MLR, SVM and RF-based classification models.

		Sensitivity	Specificity	PPV	NPV
**Morphological features**					
F0 vs F1–4	CNN	85.0%	100.0%	100.0%	96.4%
	ANN	95.0%	90.5%	70.4%	98.7%
	MLR	90.0%	98.8%	94.7%	97.5%
	SVM	95.0%	100.0%	100.0%	98.8%
	RF	95.0%	100.0%	100.0%	98.8%
F0–1 vs F2–4	CNN	80.0%	90.0%	84.2%	87.1%
	ANN	82.5%	91.7%	86.8%	88.7%
	MLR	87.5%	90.0%	85.4%	91.5%
	SVM	97.5%	100.0%	100.0%	98.4%
	RF	92.5%	91.7%	88.1%	94.8%
F0–2 vs F3–4	CNN	91.7%	97.5%	98.2%	88.6%
	ANN	100.0%	100.0%	100.0%	100.0%
	MLR	96.7%	100.0%	100.0%	95.2%
	SVM	100.0%	95.0%	96.8%	100.0%
	RF	100.0%	97.5%	98.4%	100.0%
F0–3 vs F4	CNN	96.3%	80.0%	95.1%	84.2%
	ANN	98.8%	100.0%	100.0%	95.2%
	MLR	100.0%	100.0%	100.0%	100.0%
	SVM	100.0%	90.0%	97.6%	100.0%
	RF	100.0%	95.0%	98.8%	100.0%
**Textural features**					
F0 vs F1–4	CNN	85.0%	100.0%	100.0%	96.4%
	ANN	80.0%	98.8%	94.1%	95.2%
	MLR	0.0%	98.8%	0.0%	80.0%
	SVM	100.0%	2.5%	20.4%	100.0%
	RF	95.0%	98.8%	95.0%	98.8%
F0–1 vs F2–4	CNN	80.0%	90.0%	84.2%	87.1%
	ANN	60.0%	96.0%	92.3%	78.4%
	MLR	77.5%	48.3%	90.0%	45.0%
	SVM	100.0%	16.7%	44.4%	100.0%
	RF	77.5%	88.3%	81.6%	85.5%
F0–2 vs F3–4	CNN	91.7%	97.5%	98.2%	88.6%
	ANN	1.7%	97.5%	50.0%	40.0%
	MLR	90.0%	45.0%	71.1%	75.0%
	SVM	100.0%	20.0%	65.2%	100.0%
	RF	95.0%	87.5%	91.9%	92.1%
F0–3 vs F4	CNN	96.3%	80.0%	95.1%	84.2%
	ANN	100.0%	60.0%	91.0%	100.0%
	MLR	95.0%	50.0%	88.4%	71.4%
	SVM	100.0%	55.0%	89.9%	100.0%
	RF	100.0%	95.0%	96.4%	100.0%
**All features**					
F0 vs F1–4	CNN	85.0%	100.0%	100.0%	96.4%
	ANN	100.0%	100.0%	100.0%	100.0%
	MLR	0.0%	98.8%	0.0%	80.0%
	SVM	100.0%	37.5%	28.6%	100.0%
	RF	90.0%	98.8%	94.7%	97.5%
F0–1 vs F2–4	CNN	80.0%	90.0%	84.2%	87.1%
	ANN	97.5%	98.3%	97.5%	98.3%
	MLR	77.5%	48.3%	50.0%	76.3%
	SVM	100.0%	16.7%	44.4%	100.0%
	RF	82.5%	86.7%	80.5%	88.1%
F0–2 vs F3–4	CNN	91.7%	97.5%	98.2%	88.6%
	ANN	100.0%	100.0%	100.0%	100.0%
	MLR	90.0%	45.0%	71.1%	75.0%
	SVM	100.0%	5.0%	61.2%	100.0%
	RF	100.0%	95.0%	98.8%	100.0%
F0–3 vs F4	CNN	96.3%	80.0%	95.1%	84.2%
	ANN	98.7%	100.0%	100.0%	95.2%
	MLR	95.0%	50.0%	88.4%	71.4%
	SVM	100.0%	17.6%	82.5%	100.0%
	RF	100.0%	95.0%	98.8%	100.0%

PPV: positive predictive value. NPV: negative predictive value.

### Performance of the deep learning (CNN and transfer learning)-based algorithm

For the transfer learning approach, we used a pre-trained deep learning neural network to stage the liver fibrosis samples (AUROC = 0.93 for Stage 0 vs. 1, 2, 3 and 4, AUTOC = 0.85 for Stage 0 and 1 vs. 2, 3 and 4, AUTOC = 0.95 for Stage 0, 1 and 2 vs. 3 and 4, AUTOC = 0.88 for Stage 0, 1, 2 and 3 vs. 4 (Fig. 4). We found significant differences between the transfer learning approach and the other simple learning algorithms (e.g. MLR and SVM), especially when textural features were included in the analysis. The deep learning-based algorithm enabled self-training using various combinations of filters/kernels. This learning approach was also employed in the 7-layered AlexNet-CNN algorithm during the network training process, resulting in faster and easier fine-tuning of the pre-trained network than from scratch.

### Performance of the ANN-based algorithm

The ANN-based algorithm is a simplified version of the deep neural network. Next, we investigated the relationship between complexity of the neural network structure and accuracy of the classification model. The ANN-based algorithm gave rise to accurate staging of the four stages of liver fibrosis with the use of morphological features only (AUROC = 0.92 for Stage 0 vs. 1, 2, 3 and 4, AUROC = 0.87 for Stage 0 and 1 vs. 2, 3 and 4, AUROC = 1.00 for Stage 0, 1 and 2 vs. 3 and 4, AUROC = 0.99 for Stage 0, 1, 2 and 3 vs. 4). It also accurately staged liver fibrosis using textural features (AUROC = 0.89 for Stage 0 vs. 1, 2 and 3 and 4, AUROC = 0.78 for Stage 0 and 1 vs. 2, 3 and 4, AUROC = 0.80 for Stage 0, 1, 2 and 3 vs. 4, except for Stage 0, 1 and 2 vs. 3 and 4 while AUROC = 0.50 for Stage 0, 1 and 2 vs. 3 and 4) or using both morphological and textural features (AUROC = 1.00 for Stage 0 vs. 1, 2 and 3 and 4, AUROC = 0.98 for Stage 0 and 1 vs. 2, 3 and 4, AUROC = 1.00 for Stage 0, 1 and 2 vs. 3 and 4, AUROC = 0.99 for Stage 0, 1, 2 and 3 vs. 4) (Fig. 4). Different weights were allocated to the various features depending on their contribution to model generation during the training process based on back propagation algorithms^24^. We also built multiple classification models using conventional ANN-based algorithms of 1 layer with 10 nodes and 2 layers with 20 nodes to compare against the standard test of 1 layer with 20 nodes. Details on the ROC curves and AUROC values can be found in Fig. S3.

### Performance of the MLR-based algorithm

The MLR-based algorithm accurately scored the four stages of liver fibrosis with the use of morphological features only (AUROC = 0.94 for Stage 0 vs. 1, 2, 3 and 4, AUROC = 0.89 for Stage 0 and 1 vs. 2, 3 and 4, AUROC = 0.95 for Stage 0, 1 and 2 vs. 3 and 4, AUROC = 1.00 for Stage 0, 1, 2 and 3 vs. 4). However, this algorithm failed to address the differences among various stages of liver fibrosis using textural features (AUROC = 0.51 for Stage 0 vs. 1, 2, 3 and 4, AUROC = 0.63 for Stage 0 and 1 vs. 2, 3 and 4, AUROC = 0.68 for Stage 0, 1 and 2 vs. 3 and 4, AUROC = 0.73 for Stage 0, 1, 2 and 3 vs. 4) or both morphological and textural features (AUROC = 0.51 for Stage 0 vs. 1, 2, 3 and 4, AUROC = 0.63 for Stage 0 and 1 vs. 2, 3 and 4, AUROC = 0.68 for Stage 0, 1 and 2 vs. 3 and 4, AUROC = 0.73 for Stage 0, 1, 2 and 3 vs. 4) (Fig. 4). This inaccurate staging of liver fibrosis using both morphological and textural features is because of the insufficient number of samples for model establishment and the large number of features that were being analyzed, resulting in the generation of a not-fully-ranked feature table that did not converge during model construction. This is a well-known problem for MLR-based classification models^25^. However, regularization or dimension reduction of feature numbers as well as use of the penalized method may overcome this problem.

### Performance of the SVM-based algorithm

For the SVM-based algorithm, accuracy of the classification model significantly decreased when textural features were added into the analysis (AUROC = 0.69 for Stage 0 vs. 1, 2, 3 and 4, AUROC = 0.61 for Stage 0 and 1 vs. 2, 3 and 4, AUROC = 0.55 for Stage 0, 1 and 2 vs. 3 and 4, AUROC = 0.68 for Stage 0, 1, 2 and 3 vs. 4) or when only textural features were used (AUROC = 0.51for Stage 0 vs. 1, 2, 3 and 4, AUROC = 0.53 for Stage 0 and 1 vs. 2, 3 and 4, AUROC = 0.53 for Stage 0, 1 and 2 vs. 3 and 4, AUROC = 0.68 for Stage 0, 1, 2 and 3 vs. 4), compared to the use of morphological features only (AUROC = 0.98 for Stage 0 vs. 1, 2, 3 and 4, AUROC = 0.99 for Stage 0 and 1 vs. 2, 3 and 4, AUROC = 0.97 for Stage 0, 1 and 2 vs. 3 and 4, AUROC = 0.95 for Stage 0, 1, 2 and 3 vs. 4) (Fig. 4). In general, the larger the margin, the lower the generalization error of the classifier^26^ for the SVM-based algorithm. Intuitively, data points could be well separated by the hyper-plane that has the largest distance to the nearest training-data point of any class (so-called functional margin). New examples and features mapped into that same space may result in large margins of error and thus make it more difficult for an optimal hyper-plane to be established. This may explain the decrease in system performance when textural features were added into the analysis.

### Performance of the RF-based algorithm

Similar to the ANN-based algorithm, the RF-based algorithm also resulted in accurate staging of liver fibrosis using morphological features only (AUROC = 0.98 for Stage 0 vs. 1, 2, 3 and 4, AUROC = 0.92 for Stage 0 and 1 vs. 2, 3 and 4, AUROC = 0.99 for Stage 0, 1 and 2 vs. 3 and 4, AUROC = 0.98 for Stage 0, 1, 2 and 3 vs. 4). It could also accurately score all stages of liver fibrosis using either textural features only (AUROC = 0.97 for Stage 0 vs. 1, 2, 3 and 4, AUROC = 0.79 for Stage 0 and 1 vs. 2, 3 and 4, AUROC = 0.94 for Stage 0, 1 and 2 vs. 3 and 4, AUROC = 0.89 for Stage 0, 1, 2 and 3 vs. 4) or both morphological and textural features (AUROC = 0.94 for Stage 0 vs. 1, 2, 3 and 4, AUROC = 0.85 for Stage 0 and 1 vs. 2, 3 and 4, AUROC = 0.98 for Stage 0, 1 and 2 vs. 3 and 4, AUROC = 0.98 for Stage 0, 1, 2 and 3 vs. 4) (Fig. 4). Within each RF, curvature or interaction tests for split-predictor selection was performed to calculate the importance of estimations against various features (predictors). For example, the median value of fiber length and ratio of short to long fibers were the two most prominent features for classification using morphological features only. The ratio of short to long fibers, median, and standard deviation of the magnitude of convolution over the image with Gabor filters at five scales were the three most important features for analysis using both morphological and textural features. In addition to generating highly accurate models, the RF-based algorithm could estimate the importance of each feature to the classification process (Fig. S4).

### Discussion

Histopathological examination of stained liver biopsy samples remains as the gold standard for monitoring liver fibrosis progression^21^. However, inter- and intra-observer and staining variations may generate errors in disease staging^27^. With the development of both mode-locked lasers and highly sensitive optical sensors, non-linear optical microscopy (such as those based on multi-photon excitation fluorescence and multi-harmonic generation) has made stain-free imaging-based diagnosis a feasible approach^28^. With the generation of large amounts of data using these imaging modalities, several groups have explored the use of machine learning-based algorithms for scoring liver fibrosis stages^10,11^. However, the value of these algorithms remains unclear as they are usually case-specific and not fully automated, requiring manual segmentation and feature extraction. In this study, we demonstrate that deep learning-based algorithms can automatically quantify liver fibrosis progression in a TAA-induced fibrosis rat model and score different stages of liver fibrosis with high sensitivity and specificity. The fully automated deep learning-based algorithm exhibited the same level of accuracy in scoring liver fibrosis as other semi-automated algorithms such as ANN, MLR, SVM and RF.

Classification accuracy was found to be lower for scoring early stage liver fibrosis (Stage 0–1) compared to advanced fibrosis (Stage 2–4). The accuracy of all classification models (AUC = 0.85–1.00) using only morphological features was also found to be comparable to previously reported models (AUC = 0.80–0.95)^11,22^. For MLR and SVM-based algorithms, the AUC was very much lower as the system performance was affected by insignificant features. The addition of textural features did not make any difference to the accuracy of the classification model using conventional ANN and RF-based algorithms. Using our classification models, almost all fibrosis stage comparisons had high AUROC values (most were higher than 0.85), indicating that the features employed in this study were crucial indices and largely comparable to the parameters used in the clinically accepted METAVIR scoring system.

While conventional semi-automated learning algorithms are useful for liver fibrosis scoring, the deep learning-based approach is more promising as it automatically finds features and calculates the weight of each feature through their contribution, making it less tedious and more cost-effective. Inherently, large datasets and complex training neural networks are required for deep learning-based algorithms; however, this can be addressed via the use of transfer learning from weakly or even irrelevant image sources. We show that the most studied 7-layered AlexNet-based network using transfer learning was capable of dealing with a limited dataset, achieving the same level of accuracy as conventional semi-automated learning algorithms. Deep learning-based algorithms also enable the assessment of important features; this determines the minimally required set of features for the classification, hence reducing the number of parameters^29^. Future work will include validating these models against other scoring systems such as the Ishak staging and Beijing P-I-R classification^30^ for the analysis of intra-stage cirrhosis to patient samples and validate this approach by correlating with non-invasive imaging or blood test markers. We will also consider other types of deep learning neural networks besides DCNN to find whether better performance can be achieved.

## Conclusion

Comparing different machine learning-based algorithms, we demonstrate that a deep learning-based algorithm using pre-trained AlexNet-CNN can automatically score liver fibrosis stages with a level of accuracy similar to conventional ANN, non-linear MLR and linear SVM, and feature ranking-based RF algorithms. A transfer learning approach using weakly or even irrelevant image sources may also help to address the requirement of large datasets for deep learning-based algorithms. This computer-aided and fully automated quantification of scoring liver fibrosis stages can be generalized to the design of high performance classification systems for other medical imaging tasks.

## Electronic supplementary material

Supplementary information
