# Supplementary material for: Deep learning enables automated scoring of liver fibrosis stages
Source: Sci Rep. 2018 Oct 30;8:16016. doi: 10.1038/s41598-018-34300-2 (PMC6207665; doi:10.1038/s41598-018-34300-2)
Supplement: Supplementary file 1 — Supplementary information [file 41598_2018_34300_MOESM1_ESM.docx]

**Supplementary Information**

Deep learning enables automated scoring of liver fibrosis stages

Yang Yu^1, 2, 3^, Jiahao Wang^4^, Chan Way Ng^2, 5, 6^, Yukun Ma^2, 6^, Shupei Mo^1^, Eliza Li Shan Fong^7^, Jiangwa Xing^1^, Ziwei Song^1, 2^, Yufei Xie^8^, Ke Si^4, 9^, Aileen Wee^10, 11^, Roy E. Welsch^12, 13^, Peter T.C. So^3, 14, 15^, Hanry Yu^1, 2, 3, 6, 16, 17*^

^1^Institute of Bioengineering and Nanotechnology, Agency for Science, Technology and Research (A*STAR), Singapore 138669

^2^Department of Physiology, Yong Loo Lin School of Medicine, National University of Singapore, Singapore 117597

^3^BioSystems and Micromechanics (BioSyM), Singapore-MIT Alliance for Research and Technology, Singapore 138602

^4^Institute of Neuroscience, Department of Neurobiology, Key Laboratory of Medical Neurobiology of the Ministry of Health of China, Zhejiang Province Key Laboratory of Neurobiology, School of Medicine, Zhejiang University, Zhejiang, China 310058.

^5^NUS Graduate School of Integrative Sciences and Engineering, National University of Singapore, Singapore 117411

^6^Mechanobiology Institute, National University of Singapore, Singapore 117411

^7^Department of Biomedical Engineering, National University of Singapore, Singapore 117411

^8^Duke-NUS Graduate Medical School Singapore, National University of Singapore, Singapore 169857

^9^State Key Laboratory of Modern Optical Instrumentation, College of Optical Science and Engineering, Zhejiang University, Zhejiang, China 310027

^10^Department of Pathology, National University Hospital, Singapore 119074

^11^Department of Pathology, Yong Loo Lin School of Medicine, National University of Singapore, Singapore 119074

^12^Sloan School of Management, Massachusetts Institute of Technology, Cambridge, MA USA 02139

^13^Center for Statistics and Data Science, Massachusetts Institute of Technology, Cambridge, MA USA 02139

^14^Department of Mechanical Engineering, Massachusetts Institute of Technology, Cambridge, MA USA 02139

^15^Department of Biological Engineering, Massachusetts Institute of Technology, Cambridge, MA USA 02139

^16^Confocal Microscopy Unit & Flow Cytometry Laboratory, National University Health System, Singapore 119228

^17^Gastroenterology Department, Nanfang Hospital, Southern Medical University, Guangzhou, China 510515

* Corresponding authors:

Hanry Yu (Email: [hanry_yu@nuhs.edu.sg](mailto:hanry_yu@nuhs.edu.sg); Tel: +65 6824 7000   Fax: +65 6478 9080)


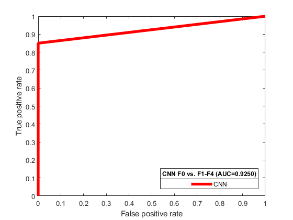

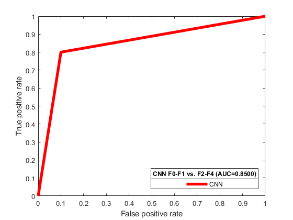

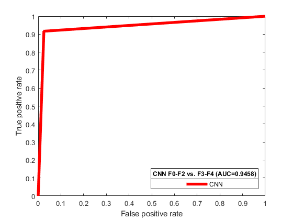

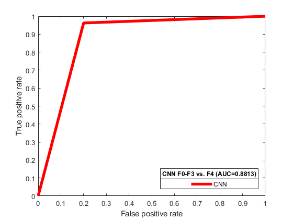


**Figure S1. Details of receiver operating characteristic (ROC) curves and corresponding area under receiver operating characteristic (AUROC) for deep learning-based classification model.**

**
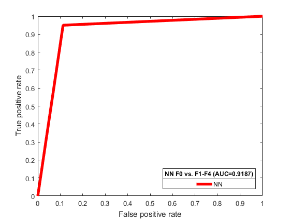

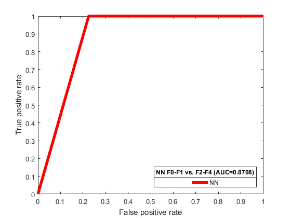

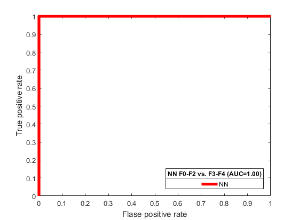

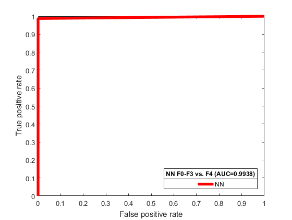
**

A

**
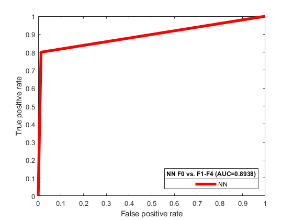

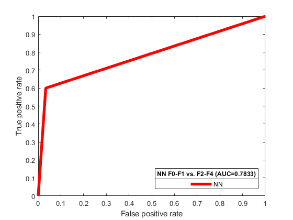

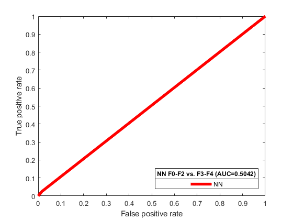

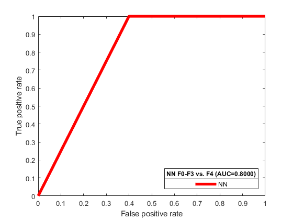
**

B

**
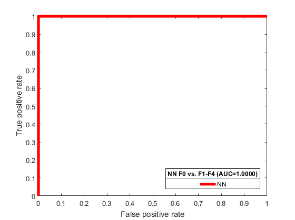

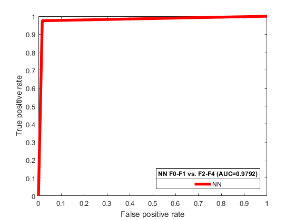

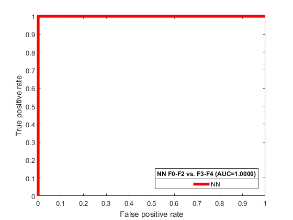

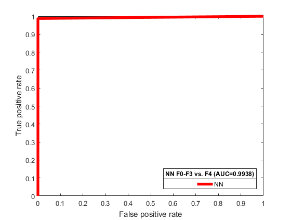
**

C

**
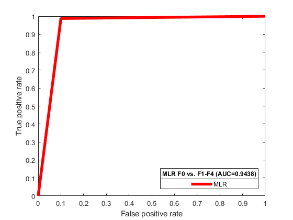

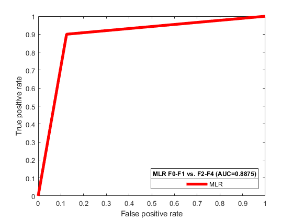

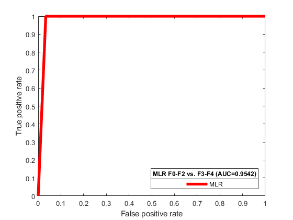

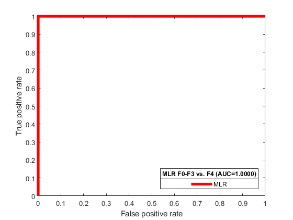
**

D

**
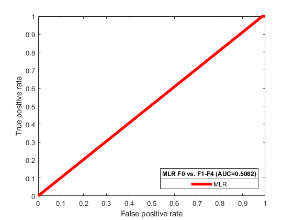

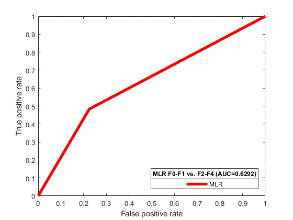

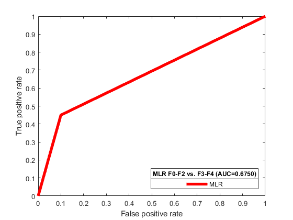

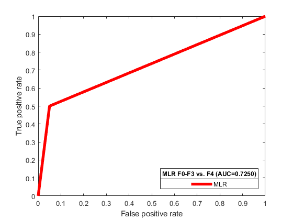
**

E

**
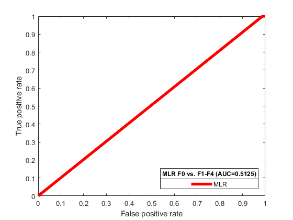

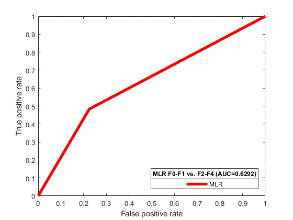

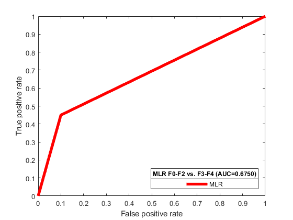

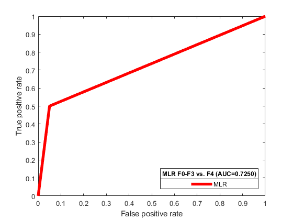
**

F

**
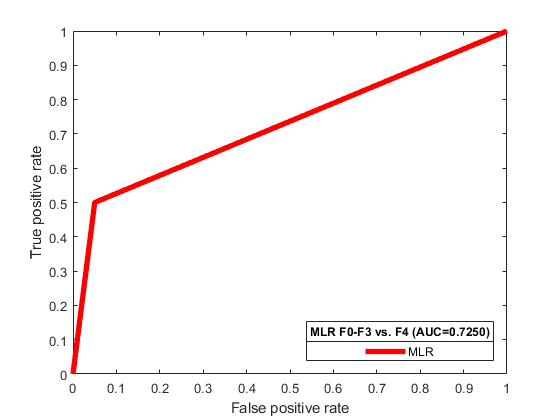
**

**Figure S2. Details of receiver operating characteristic (ROC) curves and corresponding area under receiver operating characteristic (AUROC) for non-deep learning-based classification models.** A) ROC curves and AUROC values for ANN-based classification model (1 layer, 20 nodes) using morphological features only (Features 1-21). B) ROC curves and AUROCs value for ANN-based classification model (1 layer, 20 nodes) using textural features only (Features 22-130). C) ROC curves and AUROC values for ANN-based classification model (1 layer, 20 nodes) using all features (Features 1-130). D) ROC curves and AUC value for MLR-based classification model using morphological features only (Features 1-21). E) ROC curves and AUC value for MLR-based classification model using textural features only (Features 22-130). F) ROC curves and AUC value for MLR-based classification model using all features (Features 1-130). G) ROC curves and AUROC values for SVM-based classification model morphological features only (Features 1-21). H) ROC curves and AUROCs value for SVM -based classification model using textural features only (Features 22-130). I) ROC curves and AUROC values for SVM -based classification model using all features (Features 1-130). J) ROC curves and AUC value for RF-based classification model using morphological features only (Features 1-21). K) ROC curves and AUC value for RF -based classification model using textural features only (Features 22-130). L) ROC curves and AUC value for RF -based classification model using all features (Features 1-130).


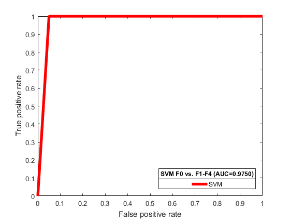

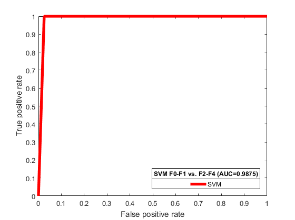

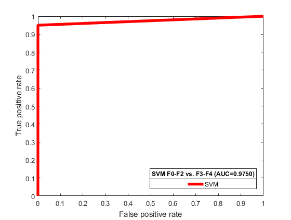

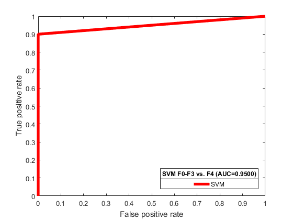


G


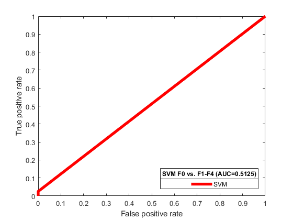

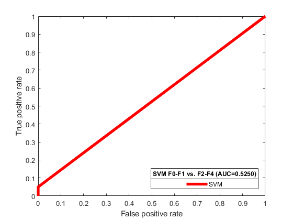

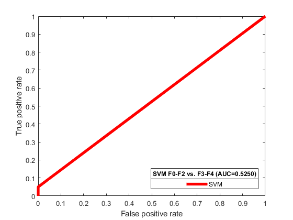

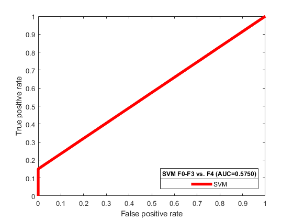


H


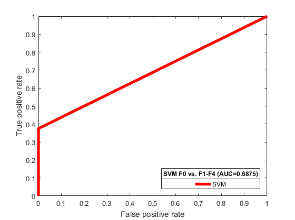

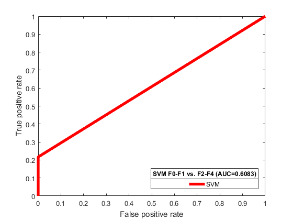

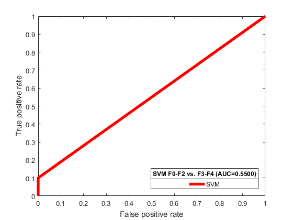

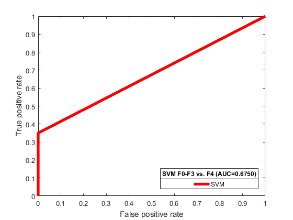


I


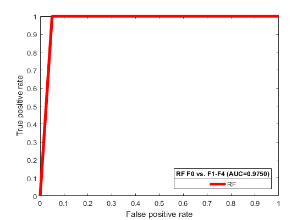

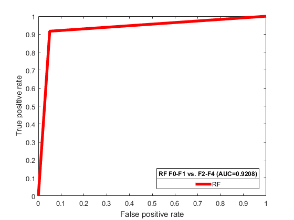

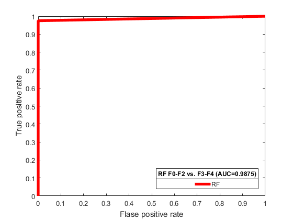

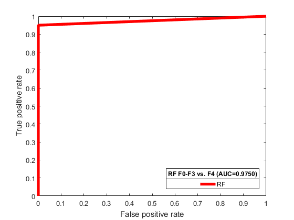


J


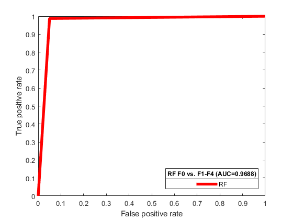

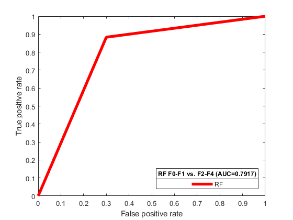

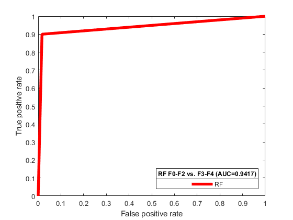

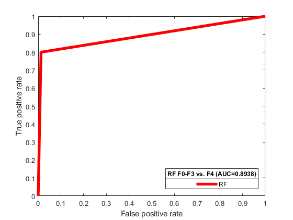


K


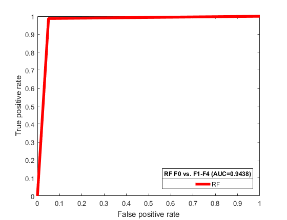

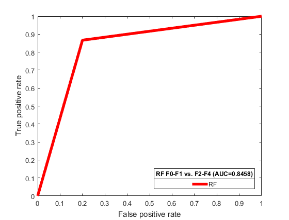

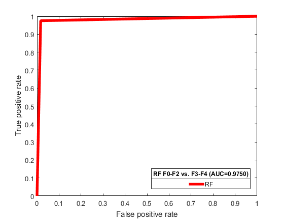

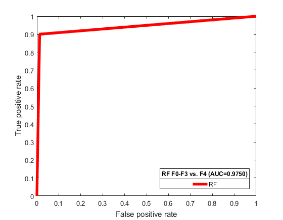


L

**Figure S2. Details of receiver operating characteristic (ROC) curves and corresponding area under receiver operating characteristic (AUROC) for non-deep learning-based classification models (continued).** A) ROC curves and AUROC values for ANN-based classification model (1 layer, 20 nodes) using morphological features only (Features 1-21). B) ROC curves and AUROCs value for ANN-based classification model (1 layer, 20 nodes) using textural features only (Features 22-130). C) ROC curves and AUROC values for ANN-based classification model (1 layer, 20 nodes) using all features (Features 1-130). D) ROC curves and AUC value for MLR-based classification model using morphological features only (Features 1-21). E) ROC curves and AUC value for MLR-based classification model using textural features only (Features 22-130). F) ROC curves and AUC value for MLR-based classification model using all features (Features 1-130). G) ROC curves and AUROC values for SVM-based classification model morphological features only (Features 1-21). H) ROC curves and AUROCs value for SVM -based classification model using textural features only (Features 22-130). I) ROC curves and AUROC values for SVM -based classification model using all features (Features 1-130). J) ROC curves and AUC value for RF-based classification model using morphological features only (Features 1-21). K) ROC curves and AUC value for RF -based classification model using textural features only (Features 22-130). L) ROC curves and AUC value for RF -based classification model using all features (Features 1-130).

N.S.

N.S.

N.S.

*

N.S.

N.S.

N.S.

N.S.

N.S.

N.S.

N.S.

N.S.

N.S.

N.S.

N.S.

N.S.

B

N.S.

N.S.

*

N.S.

N.S.

N.S.

N.S.

N.S.

C

**Figure S3. The AUROC values for various structures of conventional ANN algorithm-based classification models.** AUROC was evaluated for ANN with A) morphological features (Features 1-21), B) textural features (Features 21-130) and C) both morphological and textural features (Feature 1-130). N.S.: non-significant difference, *: adjusted p value is less than 0.017 (0.05/3).


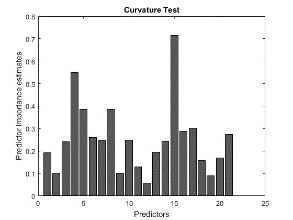

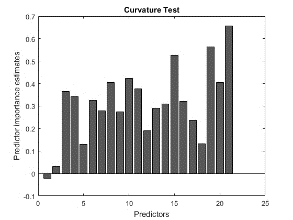

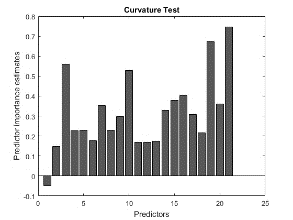

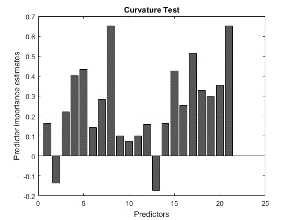


A


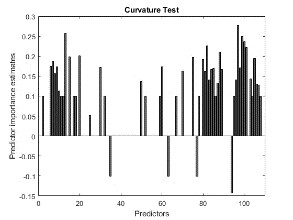

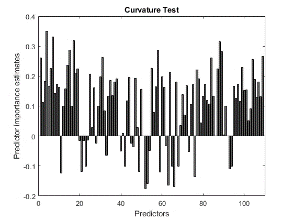

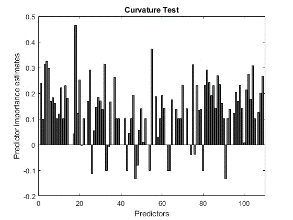

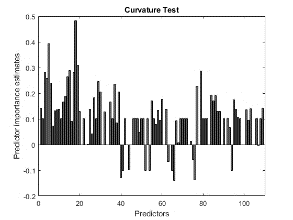


B


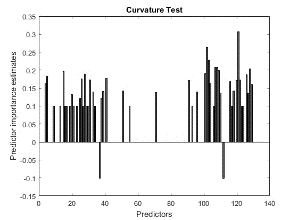

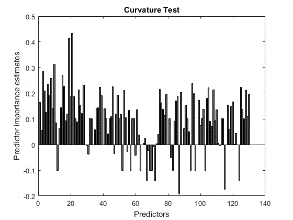

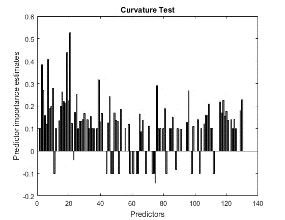

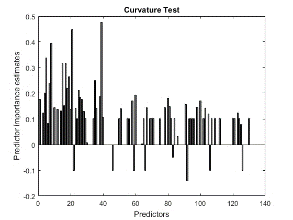


C

**Figure S4. Feature importance calculated from RF-based classification model for various comparison of fibrotic evaluation.** For those features that have higher ranking and more contribution to the classification model were highlighted by the red box. A. Random forest (feature importance) using morphological features only. B. Random forest (feature importance) using textural features. C. Random forest (feature importance) using all features.

**Table S1. Distribution of liver fibrosis score (0-4) for all the 25 TAA-induced fibrotic rats included in this study.** 4 paired biopsy slides were taken from each rat for the pathological review and Second Harmonics Generation (SHG) imaging.

| Sample name | Stage (Metavir scoring) |
| --- | --- |
| T0-1-L | 0 |
| T0-2-L | 0 |
| T0-3-L | 0 |
| T0-4-L | 0 |
| T4-1-L | 0 |
| T4-2-L | 1 |
| T4-3-L | 1 |
| T4-4-L | 1 |
| T6-1-L | 1 |
| T6-2-L | 2 |
| T6-3-L | 1 |
| T6-4-L | 2 |
| T7-1-L | 2 |
| T7-2-L | 2 |
| T7-3-L | 3 |
| T7-4-L | 3 |
| T8-1-L | 2 |
| T10-1-L | 3 |
| T10-2-L | 3 |
| T10-3-L | 4 |
| T10-4-L | 3 |
| T12-1-L | 4 |
| T12-2-L | 4 |
| T12-3-L | 4 |
| T12-4-L | 4 |
